# A Methodological Approach to Assessing Biocompatibility: 3D Printing and Subcutaneous Implantation in Rats

**DOI:** 10.1021/acsomega.5c12754

**Published:** 2026-08-04

**Authors:** Lediane Pedroso Silva, Tais da Silva, Taiana Gabriela Moretti Bonadio, Miriã Andrade de Oliveira, Karina Midori Endo, Luzmarina Hernandes

**Affiliations:** † Program in Biosciences and Physiopathology, Morphological Sciences Department, 42487 State University of Maringá (UEM), 5790 Colombo Avenue, Block H79, 87020-900 Maringá, Paraná, Brazil; ‡ Department of Physics, 307046 State University of Midwest (UNICENTRO), 838 Élio Antonio Dalla Vecchia Lane, Vila Carli Neighborhood, CEDETEG Campus, 85040-167 Guarapuava, Paraná, Brazil; § Morphological Sciences Department, State University of Maringá (UEM), 5790, Room 108, Colombo Avenue, Block H79, 87020-900 Maringá, Paraná, Brazil

## Abstract

To ensure the safety of PVDF in biomedical applications, biocompatibility tests must be performed after each manufacturing process, as these can alter the material’s properties, even if it is inherently biocompatible. Thus, this study aimed to evaluate the in vivo biocompatibility of pure PVDF and PVDF enriched with hydroxyapatite (HAp) in the subcutaneous tissue of Wistar rats and to characterize the crystalline structures of the materials. Subcutaneous implants of PVDF and PVDF-HAp were performed in Wistar rats. Tissue response was evaluated histologically using scoring at 7, 15, and 30 days, according to ISO 10993-6:2016. The crystalline structure of PVDF, HAp, and PVDF-HAp was analyzed by X-ray Diffraction (XRD). FTIR analysis was conducted to further investigate the crystalline structure. XRD analysis confirmed the presence of the α and β phases of PVDF and indicated good interaction between PVDF and HAp in the composite. The FTIR results also demonstrate that the β polar phase is predominant in printed samples. Both materials demonstrated biotolerance, as defined by the ISO standard, inducing only a mild inflammatory reaction. Based on the results, it is concluded that PVDF and the composite with HAp were biotolerable. The study demonstrates a biocompatible baseline, representing a mandatory preliminary step for future orthotopic bone evaluation.

## Graphical abstract

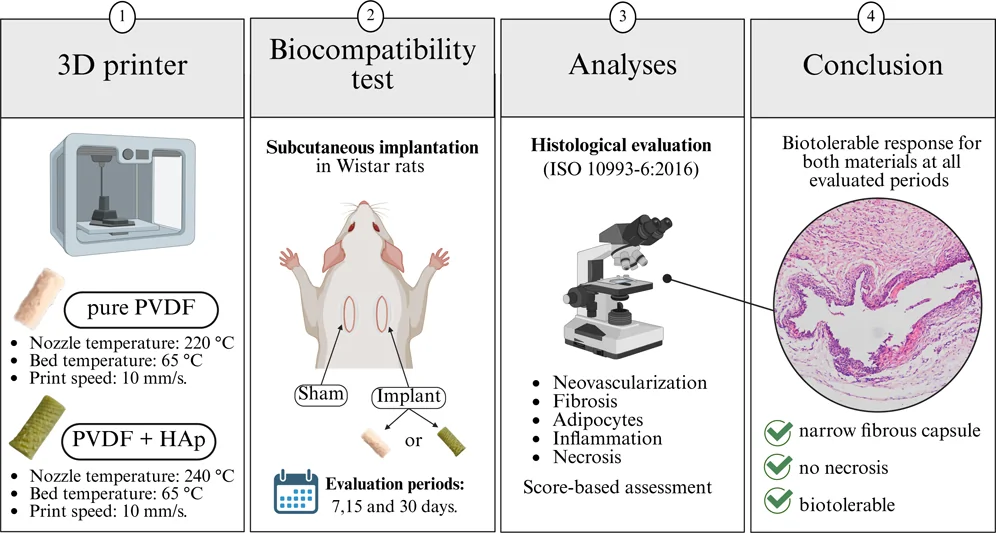

## Introduction

Tissue engineering and regenerative medicine are rapidly expanding research areas, driven by the need for novel biomedical materials^1,2^ and effective solutions for repairing and replacing damaged tissues and organs.^3,4^ The primary driving force behind technological innovation in biomaterials, implants, and medical devices is to improve patient quality of life and survival.^5^

The clinical application of these materials depends on rigorous biocompatibility and efficacy testing, which evaluates their interaction with the body and ensures patient safety. Biocompatibility tests focuses on the ability of a medical device to elicit an appropriate host response in a specific application and, its evaluation serves as a measure of the magnitude and duration of the pathophysiological mechanisms that determine such a response.^6^

Preclinical animal studies play a crucial role in this process,^7^ as many clinical device complications stem from inadequate biocompatibility.^8^

Implantation tests assess the local pathological effects induced by the material on the structure and function of living tissue.^9,10^ The analysis is performed at the macroscopic and microscopic levels,^11^ with histological analysis used to characterize various biological response parameters.^12^ The procedures, protocols, guidelines, and standards used in the in vivo biocompatibility assessment of medical devices are developed by regulatory agencies, such as the International Organization for Standardization (ISO) and the Food and Drug Administration (FDA) in the United States,^9,13,14^ among others worldwide.

This study is conducted in accordance with ISO 10993-6:2016, Biological Evaluation of Medical Devices,^12^ to assess the biocompatibility of pure polyvinylidene fluoride (PVDF) and a composite containing hydroxyapatite.

PVDF is a thermoplastic fluoropolymer that stands out for its versatility and a combination of physicochemical and mechanical properties, making it attractive for various applications, including the biomedical field.^15–17^ Its semicrystalline structure exhibits four distinct crystalline phases (α, β, γ, and δ), each imparting specific characteristics to the Polymer.^15,18–20^ The α and β phases are the most stable forms in PVDF. The α phase, most easily obtained by crystallization from the melt (liquid state), is characterized by its thermodynamic stability.^18^ The β phase, of great interest for its piezoelectric and pyroelectric properties, is employed in sensors, actuators, and biomedical devices.^19,21,22^ The conversion of the α phase to the β phase can be induced by different methods, such as mechanical stretching, electrical polarization, and the addition of fillers.^18,20,23,24^

The growing interest in PVDF for biomedical applications stems from its biocompatibility, chemical resistance, mechanical flexibility, and processability.^20,25^ These characteristics allow the fabrication of devices with different shapes and geometries, meeting the specific demands of each application.^26^ When heated, PVDF loses its rigidity and becomes ductile, making it possible to process products from this material.^27^ PVDF has already been used in the manufacture of cardiac sutures, catheters, tissue regeneration membranes, controlled drug release devices, and biomedical sensors.^15,16,28^ Studies indicate that surfaces or structures made with β-phase PVDF promote cell growth, making it a promising solution in tissue engineering and regenerative therapies.^29,30^

Other materials can be combined with PVDF to form composites. In vitro studies have shown that NCTC-929 cells exhibit high viability and low cytotoxicity when exposed to electrospun PVDF-hydroxyapatite membranes.^31^ These data highlight the potential of the composite in a biological environment, and the presence of hydroxyapatite suggests its application in bone tissue, which has intrinsic piezoelectric properties.^30,32^ However, the tissue compatibility of processed PVDF composites must first be thoroughly established in soft-tissue models before evaluating their utility in hard-tissue environments and materials developed for this purpose must be evaluated according to established standards.^33^ Although PVDF is recognized as biocompatible, its processing during manufacturing can alter its crystallinity and composition, warranting new biocompatibility tests to ensure the material’s safety after each manufacturing step.

Given this gap, the present study aims to evaluate the in vivo tissue response to 3D-printed pure PVDF and PVDF-HAp composites obtained by Fused Deposition Modeling (FDM) using a standardized subcutaneous implant model in rats, in accordance with the histological parameters of ISO 10993-6:2016. The incorporation of HAp into the PVDF matrix aims to evaluate whether the interaction between the mineral phase and the polymer during FDM processing maintains a favorable biocompatibility profile. This soft-tissue study establishes a biocompatible baseline, representing a mandatory preliminary step for future orthotopic bone evaluation.

## Experimental Section

The materials used in this study were developed in the Physics Department of the State University of Midwest (UNICENTRO - Guarapuava-PR).

### Obtaining the Materials: PVDF and HAp

Hydroxyapatite (HAp) powder was obtained from bovine bone according to the method of Miyahara et al. (2007).^34^ The bones were cleaned, dried, and calcined in a muffle furnace at 5 °C/min to 900 °C for 7 h. Using an agate pestle and mortar, the powder was separated and sieved through a 75 μm/200 mesh sieve. To remove remaining impurities, the powder was again calcined at 900 °C for 4 h. It was then ground in a high-energy mill (Retsch, PM100) for 8 h at 300 rpm using a 50 mL grinding jar and 10 mm-diameter stainless steel spheres, with a 6:1 mass ratio of spheres to powder. Subsequently, the powder was dissociated in an agate mortar and pestle. Thus, the HAp powder was ready for use. Commercial PVDF (Alfa Aesar, 99.8%) was used in powder form.

### Printing PVDF and PVDF-HAp Composite Samples

The PVDF and HAp powders were mixed in an 85:15 mass ratio.^35^ Pellets of pure PVDF and the PVDF-HAp composite were produced from the powders through molding and consolidation in a muffle furnace at 170 °C. The consolidated pellets were placed in a Filmaq 3D single-screw extruder with a 1.75 mm diameter nozzle for the production of PVDF and PVDF-HAp filaments, according to procedures reported by de Oliveira et al. (2023).^35^ The filaments were used to 3D print the biomaterials using the Fused Deposition Modeling (FDM) technique with a Creality Ender-3 printer (Shenzhen, China).

Preliminary tests were conducted to optimize the 3D printing parameters for both PVDF and PVDF–HAp filaments. Nozzle temperature (200–240 °C), bed temperature (50–65 °C), and printing speed (10–100 mm/s) were systematically varied to achieve adequate layer adhesion and dimensional stability. Optimal conditions for pure PVDF were a nozzle temperature of 220 °C, bed temperature of 65 °C, and printing speed of 10 mm/s. For the PVDF–HAp composite, the best print quality was obtained at a nozzle temperature of 240 °C, bed temperature of 65 °C, and printing speed of 10 mm/s^35^.

For the in vivo studies, PVDF and PVDF-HAp composite rods were printed with a diameter of 2.0 ± 0.1 mm and a height of 5.0 ± 0.1 mm. For structural analysis by X-ray Diffraction (XRD), disc-shaped samples with a diameter of 6.0 ± 0.1 mm and a height of 1.0 ± 0.1 mm were printed. The samples were sanded with no. 600 sandpaper and cleaned in an ultrasonic bath with alcohol, distilled water, and ultrapure water, respectively.

### Structural Characterization by XRD

XRD was used to evaluate the crystalline structure of the samples. A Shimadzu diffractometer, model XRD7000, was used with Cu K­(α) radiation, a voltage of 40 kV, and an electrical current of 30.0 mA. Measurements were performed in a 2θ range from 15 to 70° and with a scanning speed of 2°/min. The obtained results were compared with data available on the Crystal Structure Database platform (https://bdec.dotlib.com.br/) and also in scientific articles.

### FTIR Measurements

The relative fraction of the β-phase in the printed samples, after the sanding process, was determined by Fourier Transform Infrared Spectroscopy (FTIR). The calculation was performed based on the characteristic absorption bands of the α and β phases, according to the method described by Cai et al.^36^

FTIR measurements were carried out using an IRSpirit spectrometer (Shimadzu, Japan) with a spectral resolution of 4 cm^–1^. The spectra were acquired using an attenuated total reflectance (ATR) accessory (Shimadzu QATR-S). All spectra were normalized, and baseline correction was applied prior to analysis to ensure accurate comparison between samples.

### In Vivo Experiments

All procedures involving the use of animals were approved by the Ethics Committee on the Use of Animals in Experimentation of UEM (CEUA/UEM: 1207310723), constituted under the terms of Article 8 of Federal Law 11.794/2008.

Male Wistar rats (*Rattus norvegicus*), 60 days old, weighing 400–500 g, from the UEM Central Bioterium were used.

### Surgical Procedure

The procedures adopted in this study followed the guidelines of ISO 10993-6:2016. According to the standard, the subcutaneous implant method should be used to determine the biological response of subcutaneous tissue to an implanted material.

The animals were anesthetized with a combination of 2% xylazine hydrochloride (20 mg/kg animal weight) (Anasedan, Ceva, Paulínia, São Paulo, Brazil) and 10% ketamine (100 mg/kg animal weight) (Dopalen, Ceva, Paulínia, São Paulo, Brazil) in a 1:1 ratio, intramuscularly. Manual epilation of the dorsal region and cleaning with 70% alcohol were performed. The animal was positioned on a surgical table, and topical iodine was applied to the epilated region. The eyes were covered to prevent drying. Two transverse incisions were made on the dorsal region of each animal to create a subcutaneous pocket, and the tissue was dissected with the aid of scissors (in a head-to-tail direction). The base of the pocket was made more than 10 mm from the incision line. The pockets on the right side received an implant of the biomaterial. The pocket on the left side did not receive any material, representing the negative control group (sham). A simulated surgical procedure (sham) can be used to evaluate the impact of the procedure on the tissue according to ISO 10993–6:2016 standard. The incisions were sutured.

### Post-Surgical Monitoring and Collection

After recovery from anesthesia, the animals were kept in individual cages under a 12 h light/dark cycle, at 22 ± 2 °C, with 50% humidity, and with food and water *ad libitum*. The animals were observed daily. At 7, 15, and 30 days postimplantation, the animals were euthanized with an overdose of the ketamine and xylazine combination (double dose), intramuscularly.

Each implant site was examined for alterations in skin structure, such as hematoma, edema, and/or other macroscopic findings, as well as implant location. Skin samples were collected and fixed in 4% paraformaldehyde. After fixation, the samples were processed for paraffin embedding.

### Microscopic Study

Semiserial sections of 7 μm were made, and the slides were stained with Hematoxylin–Eosin for descriptive and quantitative study. The slides were analyzed using a Nikon optical microscope (Eclipse 80i, Tokyo, Japan) equipped with a high-resolution camera (Nikon DS-Fi1c, Tokyo, Japan). Specific image capture software, Nikon brand (NIS-Elements version 4.0, Prague, Czech Republic), was used.

ISO 10993-6:2016 stipulates that biocompatibility analyses be performed using scores that evaluate tissue response to the material (Tables 1 and 2). The biological response parameters evaluated include: (a) the extent of fibrosis/fibrous capsule and inflammation; (b) degeneration determined by changes in tissue morphology; (c) the number and distribution of inflammatory cells, namely polymorphonuclear cells, lymphocytes, plasma cells, macrophages, and multinucleated cells, as a function of distance from the material/tissue interface; (d) the presence and extent and type of necrosis; (e) other tissue alterations, such as vascularization, fatty infiltration; (f) adverse histological responses.

**Table 1:** Histological Evaluation System—Cell Type/Response^a^

	score				
cell types/response	0	1	2	3	4
Polymorphonuclear	0	rare, 1 to 5/phf	5 to 10/phf	heavy infiltrate	packed
lymphocytes	0				
plasma cells	0				
macrophages	0				
giant cells	0	rare, 1 to 2/phf	3 to 5/phf		sheets
necrosis	0	mínimal	mild	moderate	severe

a phf = per high powered (400×) field.

**Table 2:** Tissue Response

	Score				
response	0	1	2	3	4
Neovascularization	0	minimal capillary proliferation, focal, 1 to 3 buds	groups of 4 to 7 capillaries with supporting fibroblastic structures	broad band of capillaries with supporting fibroblastic structures	Extensive band of capillaries with supporting fibroblastic structures
Fibrosis	0	narrow band	moderately thick band	thick band	Extensive band
Fatty infiltrate	0	minimal amount of fat associated with fibrosis	several layers of fat and fibrosis	elongated and broad accumulation of fat cells about the implant site	Extensive fat completely surrounding the implant

Therefore, a scoring system was used for the histological evaluation. The system accounted for the extent of the affected area around the material. Tables 1 and 2 below represent the evaluated parameters and the adopted scores.

The number of animals analyzed varied between the groups. For the 7 days, nine animals were analyzed (*n* = 5 PVDF; *n* = 4 PVDF-HAp). Over 15 days, 10 animals were analyzed (*n* = 5 per material type). For the 30 days, eight animals were analyzed (*n* = 4 PVDF; *n* = 4 PVDF-HAp). For each animal, 10 microscopic fields were analyzed to obtain the scores. The margins and the longest axis of the rod were analyzed.

### Statistical Analysis

The Mann–Whitney U test was employed to compare the median scores between the PVDF and PVDF-HAp groups. To determine the magnitude of the effect, the Hodges-Lehmann median difference was calculated along with its 95% confidence interval (CI). Statistical significance was set at p < 0.05.

Data are presented as median and interquartile range (IQR). The IQR was defined as the interval between the 25th and 75th percentiles, providing a measure of statistical dispersion suitable for ordinal scoring data.

## Results

### Structural Characterization by XRD

Figure 1 shows the X-ray diffractograms obtained for the hydroxyapatite (HAp) powder, the printed PVDF samples, and the printed PVDF-HAp composite. The HAp diffractogram showed sharp, well-defined peaks, indicating high crystallinity of the material. No spurious phases were observed, with all peaks indexed to the hexagonal structure belonging to the *P*6_3_/*m* space group, according to the ICSD 26205 (Inorganic Crystal Structure Database) diffraction pattern.

**Figure 1:**
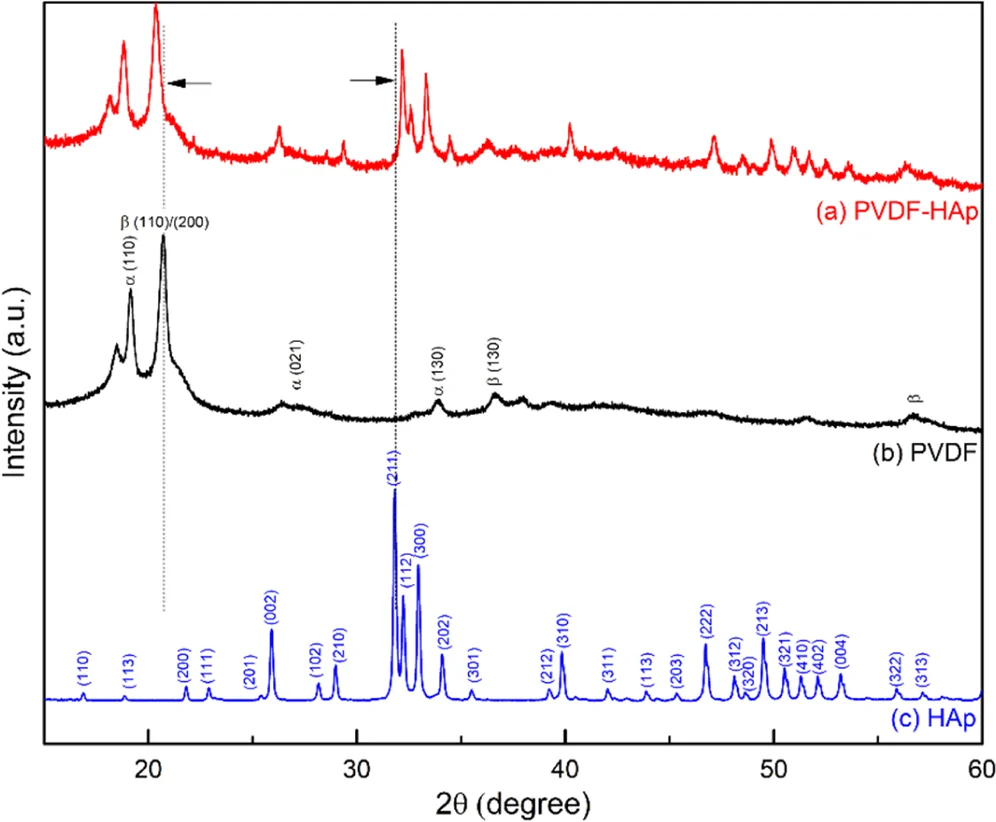
X-ray diffraction pattern referring to the HAp powder, and the printed PVDF and PVDF-HAp composite. The composite exhibited overlap between the HAp and PVDF peaks. No new crystalline phases emerged. Note the broadening of the HAp Peaks.

PVDF, being a semicrystalline polymer, presented a diffractogram characterized by broad peaks superimposed on an amorphous pattern. Nevertheless, it was possible to identify peaks attributed to the α and β phases of the polymer. The α phase was indexed to the *P*2_1_/*c* space group (monoclinic symmetry), with peaks located at 2θ = 18.41°, 19.14°, 26.35°, and 33.8°, corresponding to the crystallographic planes (020), (110), (021), and (130), respectively.^37^ On the other hand, the peaks at 20.7° and 36.3° were attributed to the (110/200) and (201) planes of the β phase, indexed to the *Cm*2*m* space group (orthorhombic symmetry).^36,37^ The peak at 56.64° can also be attributed to crystallographic plane (221) of the β phase of PVDF, according to Zhang et al.^38^

### FTIR Measurements

Figure 2 shows the FTIR spectra of the printed PVDF and PVDF-HAp. The spectra exhibit the characteristic absorption bands of PVDF, allowing the identification of α, β and γ crystalline phases of PVDF. For the PVDF-HAp composite, additional bands related to the phosphate groups (PO_4_^3–^) of hydroxyapatite can be observed. The results indicated a β-phase content of approximately 85% for pure PVDF and 84% for the PVDF-HAp composite.

**Figure 2:**
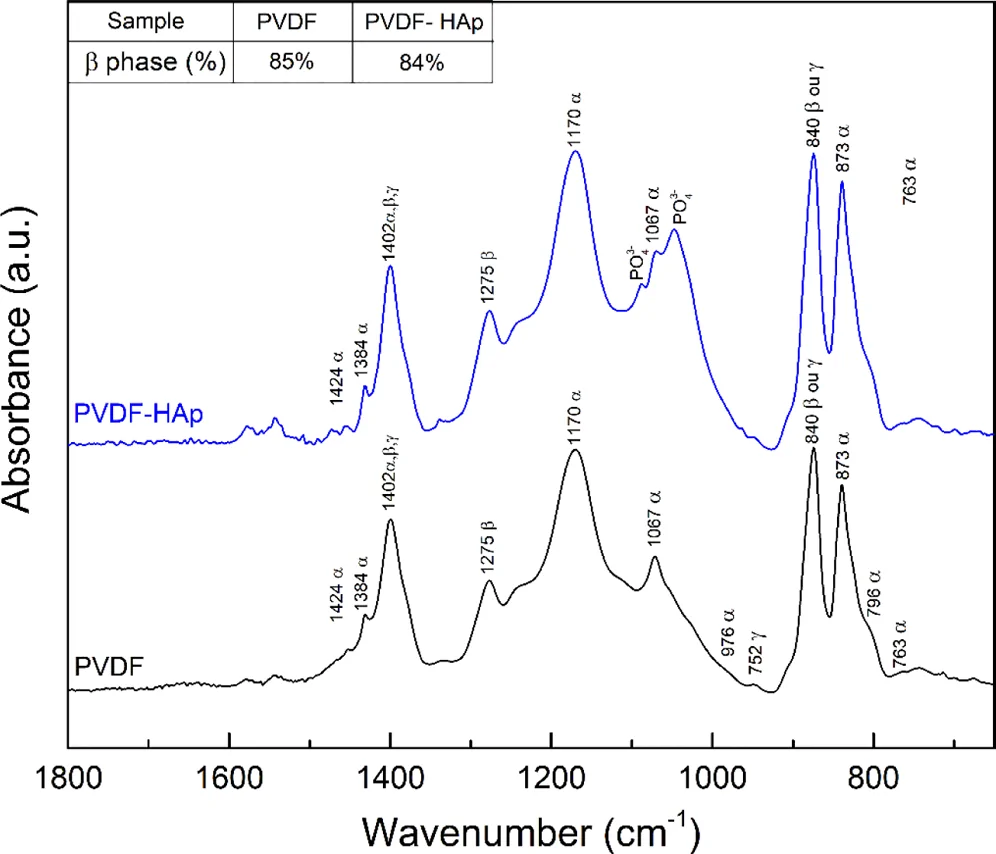
FTIR spectra of printed PVDF and PVDF-HAp samples after the sanding process. The characteristic absorption bands of the α, β, and γ phases of PVDF are indicated. The characteristic absorption bands of HAp associated with phosphate groups (PO_4_^3–^) are observed in PVDF-HAp sample. The table inset shows the calculated β-phase fraction, with values of 85% for PVDF and 84% for PVDF-HAp.

### In Vivo Studies

In this study, the animals showed normal weight gain during the experimental period compared to the beginning of the study (data not shown). Moreover, they remained clinically normal throughout the experimental period, without exhibiting signs of pain, discomfort, or behavioral changes.

Clinically, the skin in the implantation area showed mild edema and erythema. The animals maintained normal behavior throughout the analysis period (7, 15, and 30 days), with no apparent signs of pain or discomfort. The incision healing occurred without complications.

In the microscopic analysis, it was possible to observe mild inflammation, increased vascularization, development of a fibrous capsule, mild inflammatory infiltrate with the presence of polymorphonuclear cells, plasma cells, and giant cells, a minimal amount of fat associated with fibrosis, and absence of necrosis (Figure 3). These changes suggest that the evaluated biomaterials were biotolerable.

Histopathological findings were more exacerbated at the ends of the rods. In these regions, the inflammatory response, mainly characterized by the presence of polymorphonuclear cells and plasma cells, was slightly more intense. The number of vessels was also higher at the ends, and there was a greater frequency of adipocytes and multinucleated giant cells (scores of 1 to 2 in all evaluated periods) compared to the body area of the rods. The body areas were characterized by a narrow-band fibrous capsule, more consolidated, and fewer inflammatory and other cell types. These characteristics are compatible with those of biotolerable materials. No signs of necrosis were observed in any animal evaluated. Microscopic observation did not reveal any evident differences between the pure PVDF and PVDF-HAp groups. The main morphological findings are described in the captions of Figure 3.

**Figure 3:**
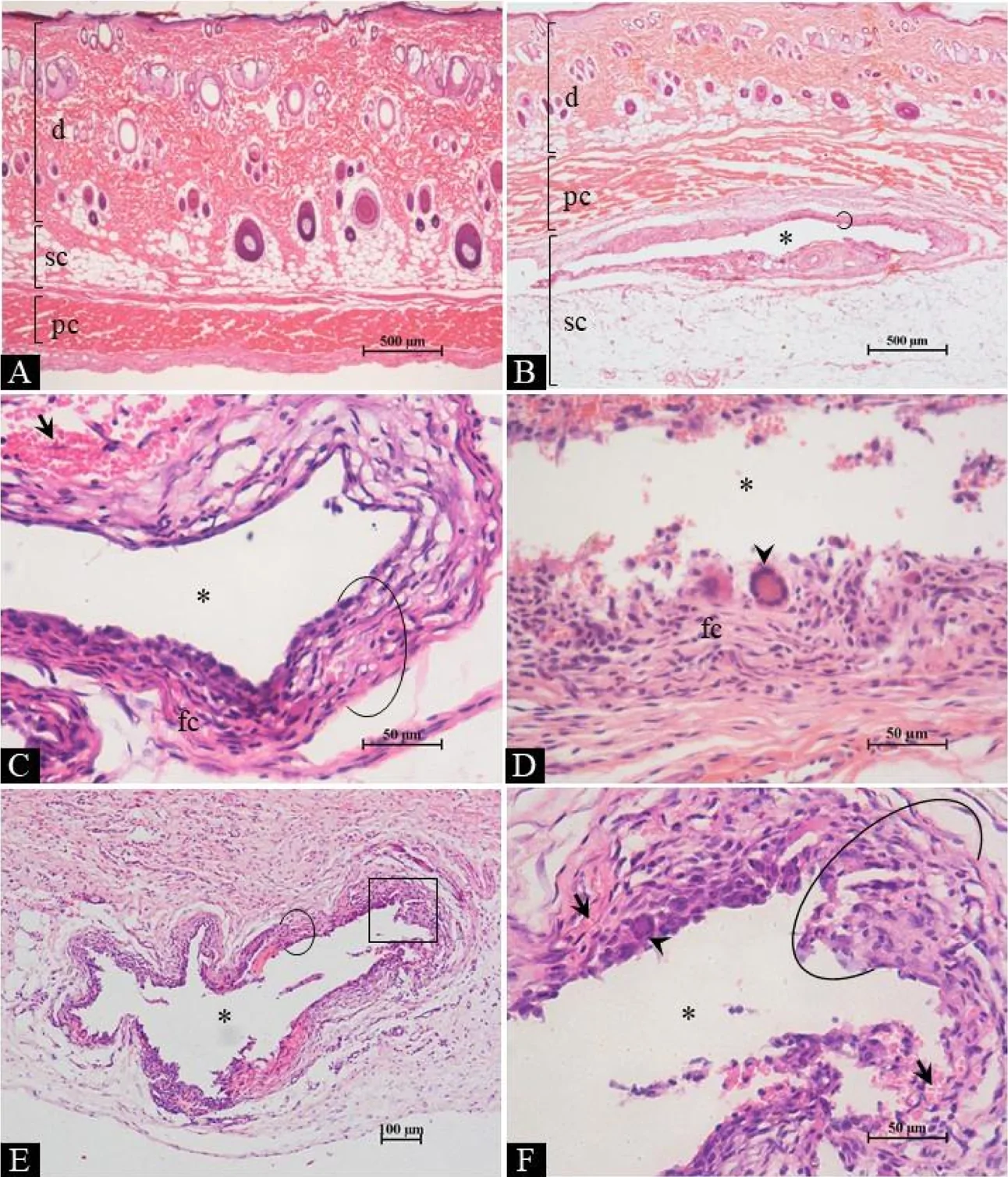
Photomicrograph of (A) normal skin. In (B–D), the biomaterial implantation site is indicated by an asterisk. In (B), PVDF-HAp implant at 7 days; in (C) and (D), pure PVDF implant at 15 and 30 days, respectively. Note the development of a fibrous capsule (fc) and circles indicate the fibrous capsule surrounding the implantation site, highlighting the tissue–material interface. Multinucleated cells are present (arrowhead). In (E,F), PVDF-HAp implant at 15 days. Image (F) is a higher magnification of the area outlined in (E), where arrows indicate the presence of blood vessels, the developing fibrous capsule (fc), and multinucleated cells. d = dermis; sc = subcutaneous tissue; pc = panniculus carnosus. Staining: hematoxylin–eosin. Original magnification: 4× (A and B); 10× (E); 40× (C,D,F).

The sham group (empty pouch) was essential to demonstrate that the inflammatory response observed in the experimental groups was associated with the implanted material and not with possible interference from the surgical procedure or the primary wound healing process. In this group, no histopathological alterations were observed.

When PVDF and the composite were statistically compared, the analyses showed that at 7 days of evaluation, the neovascularization parameter was higher for PVDF (*p* = 0,0286; Hodges-Lehmann difference = −1.000) than PVDF-HAp. The other parameters evaluated showed no statistical (Figure 4).

**Figure 4:**
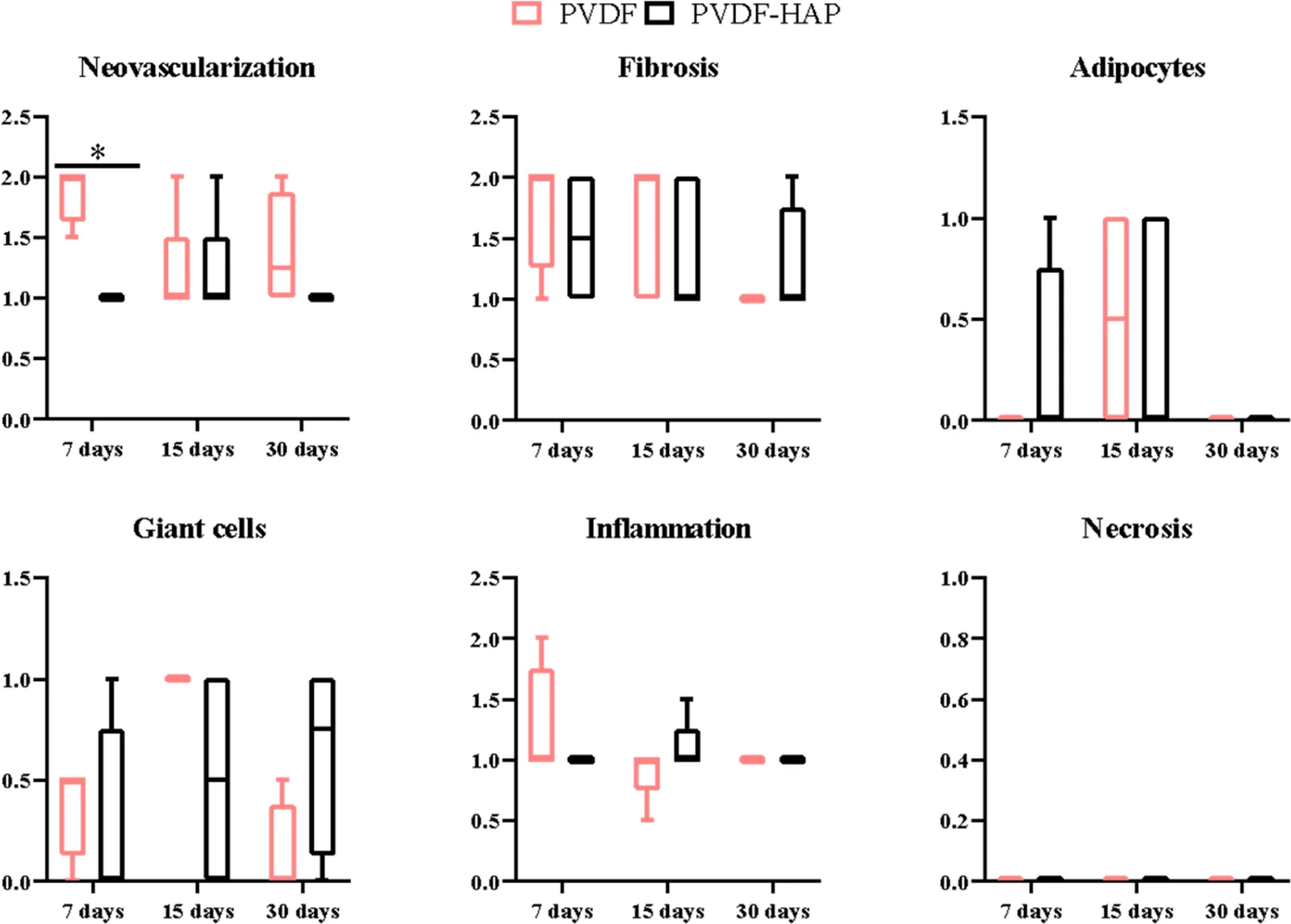
Semiquantitative histopathological scoring according to ISO 10993-6:2016 of tissue response to PVDF and PVDF-HAp at 7, 15, and 30 days after subcutaneous implantation in rats. Data expressed as Median and Interquartile Range (n = 4 to n = 5 per group/time point). Neovascularization: a significant difference was observed at 7 days, with higher scores for pure PVDF compared to the composite (p = 0.0286). The asterisk (*) indicates a statistically significant difference between material groups at the specified time point according to the Mann–Whitney U test.

## Discussion

Poly­(vinylidene fluoride) (PVDF) has emerged as a versatile polymer with applications in various fields.^39^ Its properties, such as piezoelectricity, crystalline structure, versatility, and adjustable parameters, solidify its position as a material of great interest.^40,41^ Furthermore, PVDF and its copolymers exhibit excellent processability and high thermical and chemical resistance.^22^ A particularly relevant aspect is its ability to dynamically adjust to the biological environment, favoring a more efficient interaction with nearby tissues, making it promising for the development of biomedical products.^42^

Biocompatibility refers to a material’s ability to interact with living tissues without causing adverse effects, such as cell death, hemolysis, inflammatory response, foreign body formation, or mutations.^8,43^ Assessing biocompatibility is a fundamental step in preclinical safety screening, aimed at analyzing and predicting the behavior of biomaterials intended for medical applications. This analysis ensures that the material is safe and efficient, meeting regulatory and clinical requirements for its implementation in advanced therapies.^10,44,45^

Biocompatibility is influenced by factors such as chemical composition, physical properties, the type of tissue in contact, exposure duration, and implantation site.^9,46^ PVDF is a polymer responsive to various stimuli, such as temperature, pH, humidity, light, magnetic fields, electrical fields, and mechanical pressure,^47^ allowing it to adapt dynamically within living tissues.^48^

To ensure the safety of PVDF in biomedical applications, biocompatibility tests must be performed after each manufacturing process, as these can alter the material’s properties, even if it is inherently biocompatible. High temperatures can lead to polymer degradation and the release of toxic substances.^49^ The addition of plasticizers, stabilizers, and other additives can compromise biocompatibility,^45^ and processing techniques such as extrusion, injection, and molding can introduce residual stresses and alter the material’s structure, thereby influencing its interaction with biological tissue. For this reason, new biocompatibility tests following PVDF processing are crucial to ensure the safety of the material in biomedical applications.^50,51^

Biocompatibility assessment begins with in vitro assays and culminates in in vivo (preclinical) studies, aiming to elucidate the interaction of the material with the organism, and to assess factors such as cytotoxicity, inflammatory response, tissue integration, and carcinogenic potential.^12,50^

Preclinical studies enable more detailed monitoring of device function and morphological details, frequent testing of laboratory parameters in a biological environment, and in situ observation of implants after euthanasia at desired intervals.^5^

Micromorphological analysis of tissue response after material implantation in a suitable animal model is the most reliable method for determining biocompatibility.^5,14,52^ According to Ratner (2019),^3^ biomaterials have the most significant impact when applied directly in vivo. In this context, Rosa et al.^10^ emphasize that assessing the biocompatibility of these materials is fundamental to ensuring patient safety and well-being, as well as preventing possible adverse responses.

In this study, the biocompatibility of pure PVDF samples and the PVDF-HAp composite, obtained by FDM, was evaluated. These materials can serve as raw material for the generation of biomaterials, with specific modeling, for medical applications, such as scaffolds for bone regeneration,^30,53,54^ or prosthetic meshes.^55^

Microscopic analyses were performed using a scoring system recommended by ISO 10993-6:2016, ranging from 0 to 4, where 0 indicated the absence of the evaluated parameter and 4 indicated strong occurrence.

Histological evaluation at 7, 15, and 30 days after implantation showed that pure PVDF rods or the composite with hydroxyapatite triggered a tissue response ranging from minimal to mild, as defined by ISO standards.

The materials were enveloped by a narrow-band fibrous capsule in most cases, which categorizes this material as biotolerable.^14,33,56^

This finding is consistent with previous in vivo studies involving PVDF-based biomaterials. Haase et al. (2020) reported that PVDF and related polymers induced a localized inflammatory response that evolved toward the formation of a thin and organized fibrous capsule without tissue necrosis.^58^ Similarly, Poli et al. (2020) observed satisfactory tissue compatibility of PVDF meshes implanted in rats, with limited inflammatory reaction and progressive tissue organization.^55^ The present findings therefore support the growing evidence that PVDF-based materials are generally well tolerated in vivo, even after different manufacturing processes.

Although PVDF is inherently hydrophobic, which could theoretically limit initial cellular interaction, our results showed a favorable biological response. This phenomenon can be explained by the immediate interaction between the biomaterial and the biological environment upon implantation. Implanted materials are immediately coated with proteins from blood and interstitial fluids, such as fibronectin and vitronectin. It is through this adsorbed protein layer, rather than the surface chemistry itself, that cells initially perceive and respond to foreign surfaces.^57^ This effect, combined with the presence of HAp in the composite group, may have contributed to the favorable tissue response observed in this study, although the underlying mechanisms were not directly investigated.

Among the parameters evaluated, the tissue response for PVDF and PVDF-HAp was statistically similar, for most criteria and time points. The only statistical difference was observed in neovascularization at 7 days (*p* = 0.0286), when the pure PVDF group exhibited higher vessel scores compared to the PVDF-HAp group. For all other parameters, no significant differences were found between the materials at 7, 15, and 30 days. Although the other parameters did not differ statistically, a moderate inflammatory response, according to the scoring table, was observed in the histopathological analysis in both groups.

The lower neovascularization scores in the PVDF-HAp group at 7 days, in the absence of significant differences in cell inflammatory scores, indicate a variation in early soft-tissue response between the groups. However, whether this reflects a true attenuation of the initial inflammatory response or simply a difference in local tissue remodeling requires further mechanistic study.

In the sham group (no material), no parameters were scored. The subcutaneous tissue recovered from the procedure (Figure 2A) showed no signs of tissue response. In a similar rodent subcutaneous implantation model, Haase et al. (2020) utilized PVDF as a clinical control benchmark, demonstrating that inherently biotolerable polymers typically elicit a transient, localized inflammatory reaction characterized by macrophage infiltration that successfully stabilizes into a thin, well-organized fibrous capsule without inducing necrosis. This physiological cell-to-capsule progression closely mirrors the scores obtained for both pure PVDF and PVDF-HAp in our study.^58^ Furthermore, our surgical negative control (sham group) confirmed that the residual soft-tissue scores were specific to the material interfaces rather than persistent operative trauma, thereby validating our characterization of the host response as a mild and biotolerable reaction.

ISO 10993-6:2016 provides guidelines for evaluating the biocompatibility of medical devices. It describes different degrees of tissue response (none, mild, moderate, severe),^12^ but regarding reactions to implants, it does not explicitly define what constitutes a “mild” or “moderate” reaction. Instead, the standard describes a series of histopathological effects that can be observed in tissues adjacent to an implanted medical device, and it is up to the pathologist, in conjunction with other experts, to assess the severity of these effects in the context of the biocompatibility study at hand.

Based on the results obtained and the criteria of ISO 10993-6:2016, it is possible to infer that the tissue reactions of PVDF and the PVDF-HAp composite are mild, according to the standard, and that the materials are biotolerable.

Mild reactions may include the presence of macrophages, immune system cells typically found in tissues. A slight increase in the number of these cells may indicate a minimal inflammatory response to the implant. There may also be a mild infiltrate of inflammatory cells, such as lymphocytes, in small amounts, which does not always suggest a significant adverse reaction. The formation of a thin layer of fibrous tissue around the implant, known as minimal fibrosis, is a typical response and generally considered normal.^52^ In addition, small amounts of multinucleated giant cells may be observed; these appear as a response to foreign materials, but in the absence of additional signs of inflammation, their presence is not usually concerning.^50,59^

Although the standard semiquantitative histopathological evaluation recommended by ISO 10993-6:2016 provides a reliable and universally validated platform for identifying in vivo biotolerance, we acknowledge that the phenotypic profile of the inflammatory response was not evaluated at a molecular level. Advanced characterizations, such as immunohistochemical (IHC) analysis targeting specific macrophage polarization markers (M1/M2 phenotypes) or pro-inflammatory cytokines, would further elucidate the subcellular mechanisms at the tissue–biomaterial interface. The absence of these molecular markers represents a limitation of this preliminary screening, and molecular and immunohistochemical analyses are necessary and represent a natural step when the material is tested in a bone defect (orthotopic) model.

The materials were analyzed by X-ray diffractometry (XRD). PVDF can exhibit a mixture of crystalline phases, and the proportions of each phase depend on the processing conditions.^60^ In this analysis, the alpha and beta phases of PVDF were identified. The alpha phase is more thermally stable but exhibits lower piezoelectricity than the beta phase, which is more desirable for tissue engineering applications.^61^

The formation of the β phase occurs in different stages of material processing.^62^ In this study, this phase was likely generated during extrusion in the 3D printer, where the material is simultaneously subjected to pressure and heating, and during printing, where the material is stretched and heated.^60,63,64^

The XRD analysis of the composite showed overlap of the HAp and PVDF peaks and broadening of the HAp peaks. This broadening may be related to several factors, mainly associated with the microstructure and crystalline state of HAp. One hypothesis is that during 3D printing, residual stresses may develop within the crystallites due to differences in thermal expansion coefficients between HAp and PVDF. These stresses can increase structural disorder and broaden peaks. The peak shift observed for both materials reinforces this hypothesis.

A notable feature in the XRD patterns of the PVDF–HAp composite was the systematic shift of diffraction peaks relative to those of the individual constituents. In the composite, PVDF reflections shifted toward lower 2θ values, while HAp reflections shifted toward higher 2θ values. According to Bragg’s law, such displacements indicate changes in interplanar spacing, suggesting the development of residual stresses in both phases after processing.^65^ Such shifts may be associated with residual stresses generated during processing, possibly arising from differences in thermal expansion coefficients and elastic behavior between the PVDF matrix and HAp particles during fused deposition modeling. The repeated heating–cooling cycles inherent to the printing process can promote differential thermal contraction upon solidification.^66,67^ PVDF, which exhibits a higher thermal expansion coefficient, tends to contract more than HAp during cooling. However, the presence of rigid HAp particles constrains the polymer matrix, leading to tensile residual stresses in PVDF and a consequent increase in its interplanar spacing, reflected by peak shifts toward lower diffraction angles. Conversely, the surrounding polymer matrix can impose compressive stresses on HAp particles, decreasing their interplanar spacing and shifting their peaks toward higher angles. This opposite peak displacement in the two phases is consistent with the possibility of stress redistribution at the polymer–ceramic interface. Although these results do not directly demonstrate mechanical coupling, they suggest that interactions between PVDF and HAp may occur during thermal processing.^66^ The observed structural changes and the possible presence of interfacial stresses may contribute to variations in the mechanical behavior and functional properties of the material, including its piezoelectric response, which is strongly dependent on crystalline structure and phase stability. However, further mechanical and electromechanical analyses are required to confirm these effects. In the composite, the shift occurred to the left for the PVDF peaks and to the right for the HAp peaks. This phenomenon suggests mutual interactions between the two materials during thermal processing. HAp (ceramic material) tends to expand less than PVDF (polymer) during heating in the printer and contract less when cooling. Thus, the PVDF matrix contracts more upon cooling, compressing the HAp and reducing its interplanar spacing, shifting its peaks to the right. On the other hand, HAp prevents the total contraction of PVDF, generating stresses that can increase the interplanar spacings of the polymer and shift its peaks to the left. This effect may be indicative of connectivity/interfacial contact between HAp and the PVDF matrix during thermal processing.^66^ However, structural peak shifts alone cannot confirm effective interfacial stress transfer or mechanical coupling, which must be systematically evaluated through dedicated mechanical testing. FTIR analysis was conducted to further investigate the crystalline structure. The spectra exhibit the characteristic absorption bands of PVDF, allowing the identification of alpha, beta and gamma crystalline phases. For the PVDF-HAp composite, additional bands related to the phosphate groups (PO43-) of hydroxyapatite were observed. The quantitative results indicated a beta-phase content of approximately 85% for pure PVDF and 84% for the PVDF-HAp composite. This represents a substantial increase compared to the PVDF powder used in this study (≈44%), previously reported by do Nascimento et al., 2026,^70^ highlighting the strong influence of the processing route on phase transformation. Overall, the similarity between the spectra indicates that the addition of hydroxyapatite did not significantly alter the crystalline phase composition, preserving the high content of the electroactive phase. These values demonstrate that the beta-phase is the predominant crystalline phase in both materials, which reinforces that the processing conditions, including extrusion and FDM printing, were effective in promoting its formation. This behavior can be attributed to the combined effects of thermal exposure and extensional forces during filament deposition, which promote chain alignment and favor the α → β transition. Similar trends have been reported in PVDF films subjected to uniaxial stretching, where the β-phase content increases significantly due to conformational rearrangement and alignment of polymer chains.^71^ The high beta-phase content is directly associated with the piezoelectric behavior of PVDF, a key property for the development of advanced multifunctional medical devices. This characteristic confirms the retention of the electroactive β-phase after printing, which is a desirable prerequisite for materials intended for electro-responsive biomedical applications.^42^

The association of polymers with inorganic nanofillers, such as hydroxyapatite, is a promising strategy for producing multifunctional composite materials.^72^

While hydroxyapatite is widely recognized in the literature for its osteoconductive properties in hard-tissue engineering,^47,64^ any potential bioactive or osteogenic role of the present composite remains speculative until directly tested in relevant osteogenic or bone defect models.^68,69^

## Conclusion

This study demonstrated that both pure PVDF and the HAp composite are biotolerable. According to the histological criteria of ISO 10993-6:2016, both materials induced a mild inflammatory reaction after subcutaneous implantation in rats.

XRD and FTIR analyses confirmed the presence of α and β crystalline phases in PVDF and demonstrated that thermal processing provided the desired electroactive phase in both pure PVDF and the composite. Whether these structural characteristics influence mechanical performance remains to be confirmed by future testing. The increase in β-phase content after processing indicates that extrusion and 3D printing promoted molecular alignment and stabilization of the electroactive phase β, which may contribute to the observed biological response.

Pure PVDF showed higher scores for neovascularization at 7 days compared to the PVDF-HAp composite. Unlike what is expected for implants in bone tissue, where neovascularization is a prerequisite for osteogenesis,^73,74^ in subcutaneous biocompatibility assays,^12^ vascular density is frequently associated with the magnitude of the inflammatory response and the persistence of granulation tissue.^75^ Therefore, the lower neovascularization observed in the PVDF-HAp group compared to pure PVDF should be interpreted as a more XRD and FTIR analysis confirmed the presence of α and β crystalline phases in PVDF and indicated possible interactions between PVDF and HAp during thermal processing. Stable tissue–material interface. This finding suggests that the incorporation of hydroxyapatite mitigated the initial irritative stimulus of the polymer, favoring a faster transition to the maturation and stabilization phase of the tissue–biomaterial interface,^59^ resulting in a more discrete and favorable biological response, while pure PVDF induced a more persistent foreign body reaction.

The study demonstrates the importance of conducting preclinical studies to evaluate medical devices in accordance with technical standards, such as the ISO 10993–6. Our conclusions are based strictly on this standardized histopathological screening, utilizing a semiquantitative scoring system that represents the international gold standard for initial biocompatibility assessment. Ultimately, the results obtained contribute to the development of safe, practical applications of PVDF in biomedical devices. Our findings establish a crucial biocompatible baseline in soft tissue, representing a mandatory preliminary step for future orthotopic bone evaluation.

## Supplementary Material



## Notes

The Supporting Information file contains a supplementary figure (Figure S1) supporting the findings of this study (PDF)
