# Supplementary material for: A Methodological Approach to Assessing Biocompatibility: 3D Printing and Subcutaneous Implantation in Rats
Source: ACS Omega. 2026 Aug 4;11(33):48939–49. doi: 10.1021/acsomega.5c12754 (PMC13520012; doi:10.1021/acsomega.5c12754)
Supplement: Supplementary file 1 [file ao5c12754_si_001.pdf]

A Methodological Approach to Assessing Biocompatibility: 3D Printing and Subcutaneous Implantation in Rats

Short title: Assessing PVDF biocompatibility

Lediane Pedroso Silva[1], Tais da Silva[1], Taiana Gabriela Moretti Bonadio[2], Miriã Andrade de Oliveira[2], Karina Midori Endo[2], Luzmarina Hernandes[3]* (lhernandes@uem.br)

[1] Program in Biosciences and Physiopathology, State University of Maringá (UEM), Department of Morphological Sciences, 5790 Colombo Avenue, block H79, Postal Code 87020-900, Maringá in the state of Paraná, Brazil.

[2] Program in Nanosciences and Biosciences, State University of Midwest (UNICENTRO), Department of Physics, 838 Élio Antonio Dalla Vecchia Lane, Vila Carli Neighborhood, CEDETEG Campus, Postal Code 85040-167, Guarapuava in the state of Paraná, Brazil.

[3*] State University of Maringá (UEM), Department of Morphological Sciences, 5790, room 108, Colombo Avenue, block H79, Postal Code 87020-900, Maringá in the state of Paraná, Brazil.

*Corresponding author: Luzmarina Hernandes
Phone number: (55) 44 3011 4706
e-mail: lhernandes@uem.br

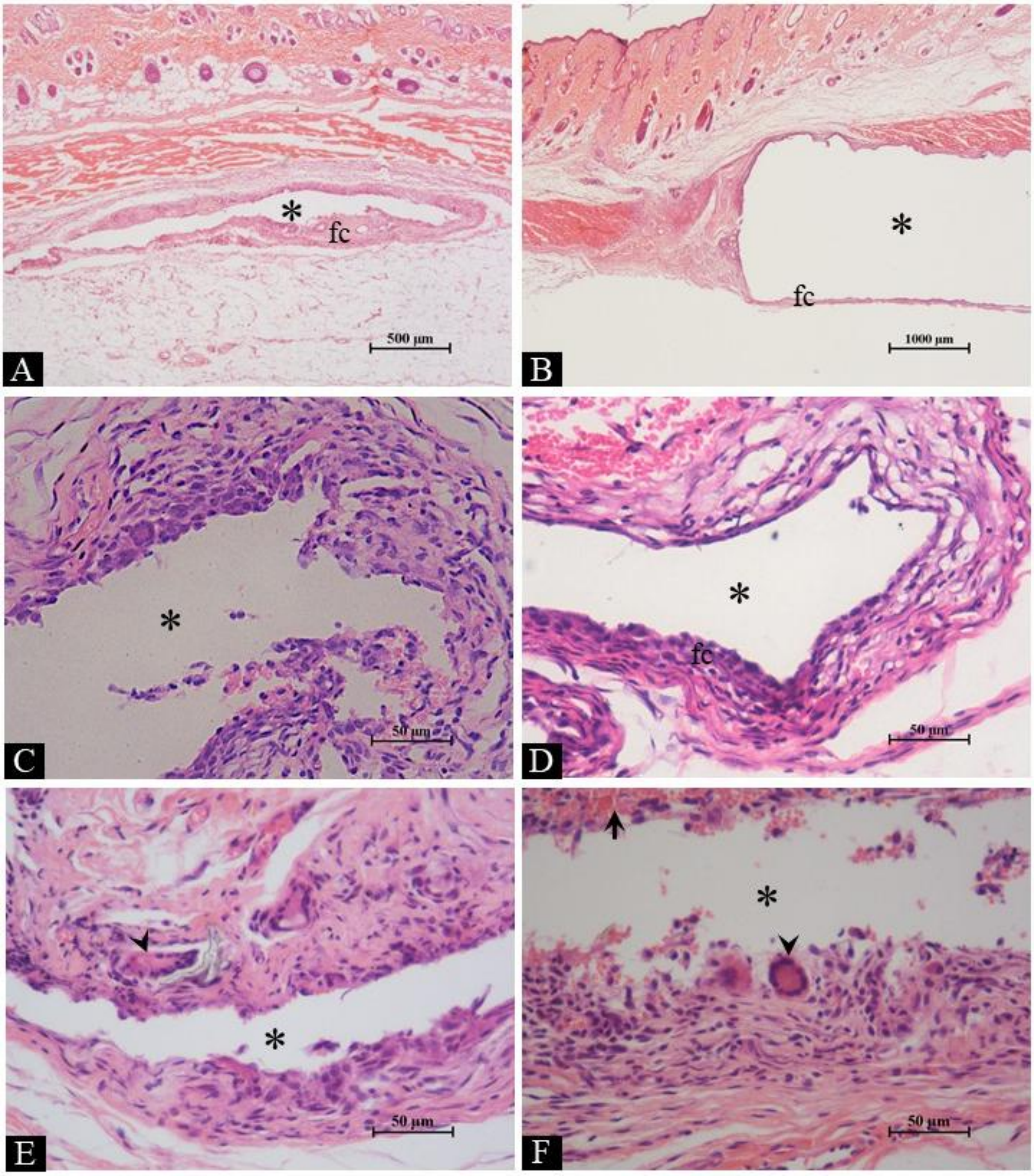

Figure S1. Photomicrographs of the subcutaneous implantation site of PVDF-HAp rods (A, C, and E) and pure PVDF rods (B, D, and F). The area occupied by the biomaterials is indicated by an asterisk (*). Panels A and B represent the implantation site at 7 days; C and D at 15 days; and E and F at 30 days. The images were arranged side by side during the same experimental period for comparative observation fc = fibrous capsule; arrowhead = multinucleated giant cell. Staining: hematoxylin and eosin (H&E). Original magnification: 4X (A), 2X (B), 40X (C,D,E and F).
